# Association between Birth Plan Use and Maternal and Neonatal Outcomes in Southern Spain: A Case-Control Study

**DOI:** 10.3390/ijerph18020456

**Published:** 2021-01-08

**Authors:** Pedro Hidalgo-Lopezosa, Ana María Cubero-Luna, Andrea Jiménez-Ruz, María Hidalgo-Maestre, María Aurora Rodríguez-Borrego, Pablo Jesús López-Soto

**Affiliations:** 1 Instituto Maimónides de Investigación Biomédica de Córdoba (IMIBIC), 14004 Córdoba, Spain; z92culua@uco.es (A.M.C.-L.); z72jirua@uco.es (A.J.-R.); en1robom@uco.es (M.A.R.-B.); n82losop@uco.es (P.J.L.-S.); 2 Departamento de Enfermería, Farmacología y Fisioterapia, Universidad de Córdoba, 14004 Córdoba, Spain; n12himam@uco.es; 3 Hospital Universitario Reina Sofía, 14004 Córdoba, Spain

**Keywords:** birth plan, natural childbirth, newborn, maternal and child health

## Abstract

Background: Birth plans are used for pregnant women to express their wishes and expectations about childbirth. The aim of this study was to compare obstetric and neonatal outcomes between women with and without birth plans. Methods: A multicentre, retrospective case–control study at tertiary hospitals in southern Spain between 2009 and 2013 was conducted. A total of 457 pregnant women were included, 178 with and 279 without birth plans. Women with low-risk gestation, at full-term and having been in labour were included. Sociodemographic, obstetric and neonatal variables were analysed and comparisons were established. Results: Women with birth plans were older, more educated and more commonly primiparous. Caesarean sections were less common in primiparous women with birth plans (18% vs. 29%, *p* = 0.027); however, no significant differences were found in instrumented births, 3rd–4th-degree tears or episiotomy rates. Newborns of primiparous women with birth plans obtained better results on 1 min Apgar scores, umbilical cord pH and advanced neonatal resuscitation. No significant differences were found on 5 min Apgar scores or other variables for multiparous women. Conclusions: Birth plans were related to less intervention, a more natural process of birth and better outcomes for mothers and newborns. Birth plans can improve the welfare of the mother and newborn, leading to birth in a more natural way.

## 1. Introduction

In today’s society, the entire process of pregnancy, childbirth and breastfeeding has been medicalised. The development of medicine has brought benefits and a decrease in maternal and infant mortality, but the use of drugs and other medical interventions in pregnant women have predisposed them to unnecessary practices. In the 1980s, a humanisation-oriented mobilisation began in childbirth care, which questions medical intervention and its adverse effects on the birth process [1].

In 1985, the World Health Organization (WHO) was urged to review the pregnancy and childbirth biomedical care model, characterised by increasing interventionism and medicalisation in developed countries. The WHO presented recommendations highlighting the importance of respecting the normal course of these processes, limiting interventions to cases supported by scientific evidence. Some of the most important recommendations (among others) were: (i) the caesarean section rate should not exceed 10–15%, as there is no justification for exceeding this rate; (ii) electronic foetal monitoring should not be routine; (iii) systematic use of episiotomy and artificial early amniotomy are not justified; (iv) vaginal deliveries after a caesarean section should be encouraged to avoid a repeat caesarean section; (v) women must be involved in decisions about their childbirth process; and (vi) breastfeeding should be established immediately after birth, before the mother leaves the delivery room [2].

In Spain, the “Normal Childbirth Care Strategy” of the National Health System, approved in 2007, is driving a profound transformation in the delivery care model. The new model considers birth as a physiological process and tries to offer personalised and comprehensive care to women based on scientific evidence, respectful of the leading role and the right to information and informed decision-making that the legislation recognises [3]. Concretely, the second strategic line of the birth plan, on the participation of women users in the decision-making process, indicates: “Promote the preparation and care of the birth and birth plan. Supporting the development of the delivery and birth plan guarantees and makes visible the dialogue between professionals and users. It is the instrument that channels the feelings of each woman.”

Therefore, this strategy includes the birth plan as a tool that can contribute to reduced labour intervention. This plan is a written document that a pregnant woman and her partner prepare before birth and use to express their wishes and expectations regarding the development of the birth process [4]. It provides guidance to the team of professionals responsible for their care during the hospital stay [5]. In addition, it serves to improve the woman’s satisfaction, promote participation in the birth process and allow her to make informed decisions. However, birth plans may be inflexible, unrealistic and can lead to conflicts and negative experiences that could affect obstetric outcomes [6,7].

The most frequently requested requirements of the birth plan are to avoid oxytocin use, episiotomy and a caesarean section, permit the ingestion of liquids, freedom of movement, intermittent monitoring, a comfortable expulsive position, immediate contact with the newborn, early breastfeeding and other conditions [8,9]. 

There are only a few studies that relate birth plan use with results associated with the mother and newborn. Some authors showed a lower percentage of caesarean sections in women who presented birth plans [10,11]; however, other authors found no significant difference between women with or without birth plans [12,13]. In addition, some authors have associated greater intervention in labour with negative outcomes in the mother and/or newborn; for example, many authors consider that intervention with oxytocin, especially at high doses or if used inappropriately, can produce negative effects as a result of uterine hyperstimulation, with possible consequences in the foetus [13].

Some parallelism could be established between the care for women with a birth plan and women enrolled in birth or alternative centres, where low-risk births are attended to in women who wish to give birth in a more intimate and different environment. In these centres, the professionals are mostly midwives who provide care based on a model of individual and family awareness and consider the birth process physiologically and naturally [14]. Since birth centres do not exist in Spain, women are admitted to hospitals to give birth; however, there is a percentage of women who demand physiological labour activity and more control of their process through birth plans. The current study research has an objective to determine whether the use of birth plans (a more natural and physiological birth process with more active attitudes of women) is associated with better maternal and neonatal outcomes.

## 2. Materials and Methods

This is a multicentre case–control study conducted at several tertiary hospitals of the Andalusia Health Public System with women who attended for birth between 2009 and 2013. The case group is composed of women who presented a birth plan document. The control group is composed of women who did not present a birth plan and gave birth with standard care. 

This study was conducted at four tertiary public hospitals with the greatest coverage in each province. In the study period, the estimated number of deliveries was 60,000 in the 4 hospitals. The sample size was determined by power analysis using EpiData software version 3.1 (Epidata, Buenos Aires, Argentina), with the following data considered: 1–2 controls per case, 29% exposure between controls, 16% exposure between cases, 80% statistical power and a confidence interval of 95%. A total of 500 women were estimated as statistically significant. Data were collected directly from medical records selected using systematic random sampling.

Inclusion criteria were established for both groups: low-risk pregnancy at term (37–42 weeks). Those excluded were women with high-risk gestation, elective caesarean birth or emergency caesarean without labour, multiple gestations, out-of-hospital birth or women who gave birth in hospitals different to those studied and birth occurring before 37 weeks of gestation. 

Sociodemographic, obstetric and neonatal variables were analysed between the two groups using SPSS statistical software (version 19; IBM Corporation, Armonk, NY, USA). Sociodemographic variables were maternal age (years), education level (primary/secondary/university studies), employment (professional activity carried out outside the domestic environment) and marital status (married/stable partner/single). Obstetric variables were parity (primiparous/secondary/≥3), gestational age (weeks), onset of labour (spontaneous/induced), epidural analgesia (yes/no), oxytocin use (yes/no), early amniotomy practice (yes/no), meconium, intrapartum maternal fever (yes/no), monitoring type (intermittent/external/internal), duration of first phase (hours), duration of second phase (minutes), type of birth (vaginal/caesarean), 3rd–4th-degree tears (yes/no) and episiotomy (yes/no). Neonatal variables were 1 min and 5 min Apgar scores, umbilical cord artery blood pH (<7.20 and mean) and neonatal advanced resuscitation (presence or not).

For data analysis, we used a statistical test of hypothesis contrast, according to the type of variable. A 95% alpha error was assumed. In the data analysis corresponding to result variables, *p*-values were adjusted using Fisher’s method. Bilateral contracts were performed using the chi-square statistic, Fisher’s exact test and Student’s *t*-test. Quantitative variables were described based on the mean value and standard deviation (SD). Analysis data considered the parity due to its ability to significantly influence the results. 

The ethics committees of the hospital centres studied gave their approval to carry out the study. This study was conducted in accordance with the ethical standards of the Helsinki Declaration (2013 review), the Council of Europe on Human Rights and Biomedicine, the UNESCO Universal Declaration on the Human Genome and Human Rights and the Oviedo Council on Human Rights and Biomedicine and after formal approval from the Ethics Committee for Research. All data were processed with confidentiality and with no third-party unauthorised access, as established under current legislation: Organic Law 15/1999 of 13 December on the Protection of Personal Data, Royal Decree 994/99 of 11 June approving the regulations on security measures for automated files that contain personal data and Organic Law 3/2018 of 5 December on the Protection of Personal Data and Guarantee of Digital Rights, a realignment of Spanish law, according to Regulation (EU) 2016/679 of the European Parliament and of the Council of 27 April, 2016 with regard to the processing of personal data and the free movement of such data. 

## 3. Results

Of the 500 women initially included in the study sample, 43 were excluded. Of those 43 exclusions, 12 had elective caesareans, 11 had emergency caesarean without labour, 12 experienced premature birth and eight had high-risk pregnancies. The final sample comprised 457 women (*n* = 457), with 178 cases or women who presented a birth plan, and 279 controls or women who did not present a birth plan document, and therefore, received standard care. 

Sociodemographic data (Table 1) show an average age of 31.27 ± 5.02 (mean ± SD) years. The minimum age was 18 years and maximum 45 years. In the case group, the average age was significantly higher than the control group (33 vs. 30 years, *p* < 0.001), and the differences were significant in nulliparous and multiparous women. The percentage of women with university-level education was significantly higher in the birth plan group than the control group (49.3% vs. 15.7%, *p* < 0.001). In addition, there was a higher percentage of women who were salaried employees in the birth plan group (78% vs. 61.7%, *p* = 0.001). 

Regarding obstetric variables (Table 1), the percentage of women who gave birth at 40 weeks or later was greater in the case group than the control group (specifically, at 41 or more weeks, 27% in the case group in comparison with 19% in the control group, *p* = 0.036).

The proportion of women who used epidural analgesia was higher in the control group than in the birth plan group (80.3% vs. 69.7%, *p* = 0.009); these differences were more significant among primiparous women. Early amniotomy was practised in 55.6% of the control group women versus 34.3% in the case group (*p* < 0.001). Oxytocin use was significantly higher in the control group, both in primiparous and multiparous women (42.6% vs. 55.1%, *p* = 0.010). The length of the first phase of birth was significantly higher in the birth plan group, with a duration of 6.28 ± 3.60 (mean ± SD) h compared to 5.20 ± 3.31 h in the control group. The length of the second phase, however, did not show significant differences between both groups.

In regard to birth results (Table 2), the percentage of caesarean sections was significantly higher in the control group among primiparous subjects (18% vs. 29%, *p* = 0.023); however, no significant differences were found among multiparous women. In comparison, there were no significant differences in instrumented births, 3rd–4th-degree tears and episiotomy in either primiparous and multiparous women. 

Neonatal results (Table 2) showed important findings at 1 min Apgar scores ≤7 (8.1% in the birth plan group versus 20.6% in the control group, *p* = 0.010), in umbilical cord arterial blood pH < 7.20 (8.7% in the birth plan group versus 21.2%, *p* = 0.011) and neonatal advanced resuscitation (4% in the birth plan group versus 15.9%, *p* = 0.008). No significant differences were found among multiparous women.

## 4. Discussion

Regarding the sociodemographic results, the data show some parallelisms with other studies. Several authors found that the ages of women who presented birth plans were higher than those who did not [10,15], and they also had better academic education levels and employment [15,16]. In general, women with a birth plan were older, primiparous and more highly educated [17]. There seems to be a relationship between higher socioeconomic status and greater interest in better birth preparation and the use of the birth plan because greater preparation allows women to take a greater interest in using the birth plan as a tool that influences the birth process, enhancing women’s safety, effectiveness, satisfaction and empowerment [10], as well as greater sense of control and protagonism, better obstetric and neonatal outcomes and a higher satisfaction [7].

Analgesia preferences are often some of the most frequent requests in birth plans. As in other studies, women with birth plans are more likely to reject epidural analgesia [18]; however, in this study, 75.4% of primiparous women used this type of analgesia. The proportion of primiparity and induction was higher in the birth plan group, although oxytocin use was significantly lower (42.6% versus 55.1%), in accordance with other studies [9,15]. One of the most important issues in birth plan documents is the use of oxytocin. In this study, 100% of participants did not want oxytocin infusion [9,15]. In general, the longer duration of the first phase of birth in women of the birth plan group (6.2 versus 5.2 h) may be due to the lack of use of oxytocin and, hence, less intervention. It is common knowledge that oxytocin and early amniotomy are associated with a shorter duration of labour [13,19].

The neonatal results of this study were found to be relevant, as suggested by the results on newborns of primiparous women, in which the variables of 1 min Apgar scores, umbilical cord blood pH values and neonatal resuscitation had better results in the birth plan group subjects. Although few studies examining neonatal results and birth plan use exist, and the authors did not find significant differences in Apgar scores [15,20], a previous study by this research group found differences in umbilical cord pH values, with better results in newborns of mothers with birth plans [15].

To our knowledge, there are few studies that relate the use of birth plans to maternal and newborn outcomes (this aspect being the main novelty of the present study). Many studies focus on assessing maternal satisfaction, birth experience or other related aspects. For this reason, data from this study were compared with data obtained from birth or alternative centres, where midwives attend births naturally and with low interventional care. In this context, some authors compared umbilical cord blood pH [21] and caesarean section rate [22] and found better results in birth centres compared to hospitals. Other authors agree that neonatal and perinatal outcomes are not worse in alternative centres, compared with those occurring in hospitals [23]. A previous study by this research group found that the greater the compliance with the birth plan, the better the results for the mother and child [24]. In fact, according to recent administrative data from the studied hospitals, adherence remains low (8–10%), although it has experienced a slight but steady increase since initiation. Therefore, professional support is essential to improve the fulfilment and compliance of birth plans, as well as the women’s adherence to the birth plans [7,24].

Another important result in this study is the reduction of caesarean births in the birth plan group women, in agreement with similar results that were found by other authors [11], who concluded that women with a birth plan were less likely to undergo a caesarean section than women without a birth plan. Indeed, Suarez-Cortés et al. found a higher percentage of normal deliveries in the group of women who presented a birth plan [10]. Other authors, however, did not find significant differences in caesarean rate between both groups [12,25]. Similarly, no differences were found in other previous studies carried out by the research group, showing data limited to women’s low adherence to birth plans and in a single centre study [15].

Although our study shows data from previous years, it is no less true that recent information on the implementation of the birth plan corroborates that there is still a low implementation at present and, on the other hand, clarifies that the birth plan has benefits for both the mother and the newborn, that it has low adherence and that it is important to encourage its use.

These results must be viewed with caution due to the limitations of this study. First, the heterogeneity of professionals who attend births may influence the degree of compliance with the birth plan. This diversity has been observed in all hospitals where this research was performed. Second, data were collected from four hospitals, corresponding to four provinces of Andalusia (in total, there are eight provinces).

## 5. Conclusions

In conclusion, our study results suggest that birth plans are used only by a minority of women giving birth. These women tend to be older, better educated and have a higher employment rate. In addition, women with birth plans had a higher rate of primiparity, induction of labour and experienced a longer mean duration of the first phase of labour. They also required less use of oxytocin, early amniotomy, epidural analgesia and general monitoring. The results obtained, both obstetrical and neonatal, were better in primiparous women.

In clinical practice, the results of this study can be of interest to professionals in this area. Birth plans can be utilised in women as informed consent to obtain a more natural birth process and could be an effective tool not only in achieving better satisfaction and birth experience, as some studies have shown, but also in obtaining better results for the mother and newborn. There are some important challenges related to birth plan use. Important efforts must be made to raise awareness of women during pregnancy (including family) and healthcare professionals. The midwife is the professional who must be present with the pregnant woman in the preparation of the document. Further research is necessary regarding the benefits of birth plan use on maternal and child health and how the knowledge acquired by women can influence subsequent pregnancy.

## Figures and Tables

**Table 1:** Sociodemographic and obstetric characteristics of women with birth plans (case group) and without birth plans (control group).

Variable	Case Group	Control Group	
	(*n* = 178)	(*n* = 279)	
	*n* (%)	*n* (%)	*p*-Value
Age ^a^	33 ± 4.32	30.17 ± 5.13	<0.001
Education level ^b^			
Primary	35 (23.3)	156 (59.8)	
Secondary	41 (27.3)	64 (24.5)	<0.001
University	74 (49.3)	41 (15.7)	
Employment ^b^	117 (78)	161 (61.7)	0.001
Marital status ^c^			
Married	85 (61.6)	180 (70.3)	
Stable partner	50 (36.2)	65 (25.4)	0.056
Single	3 (2.2)	11 (4.3)	
Parity			
Primiparous	134 (75.3)	158 (56.6)	
Secondiparous	37 (20.8)	95 (34.1)	<0.001
≥3	7 (3.9)	26 (9.3)	
Gestational age (weeks)			
37–39 + 6	66 (37.1)	135 (48.4)	
40–40 + 6	64 (36)	91 (32.6)	0.036
>41	48 (27)	53 (19)	
Onset of labour			
Spontaneous	130 (73)	226 (81)	0.045
Induction	48 (27)	53 (19)	
Epidural analgesia	124 (69.7)	224 (80.3)	0.009
Primiparous	101 (75.4)	143 (90.5)	0.001
Multiparous	23 (52.3)	81 (66.9)	0.084
Oxytocin ^d^	75 (42.6)	152 (55.1)	0.010
Primiparous	66 (50)	100 (63.3)	0.023
Multiparous	9 (20.5)	52 (41.1)	0.006
Early amniotomy	61 (34.3)	155 (55.6)	<0.001
Meconium in amniotic fluid ^e^	23 (14.3)	35 (17.9)	0.352
Intrapartum maternal fever ^f^	9 (6)	27 (10.5)	0.127
Monitoring ^g^			
Intermittent	42 (24.3)	1 (0.4)	
External	117 (67.6)	219 (83.6)	<0.001
Internal	14 (8.1)	42 (16)	
Duration 1st phase ^a^ (hours)	6.28 ± 3.60	5.20 ± 3.31	<0.001
Duration 2nd phase ^a^ (minutes)	39.66 ± 23.80	39.67 ± 25.20	0.997

^a^ Mean ± SD, ^b^ *n* = 411, ^c^ *n* = 394, ^d^ *n* = 452, ^e^ *n* = 356, ^f^ *n* = 406, ^g^ *n* = 435.

**Table 2:** Obstetric and neonatal results of women with and without birth plans.

PRIMIPAROUS (*n* = 292)				MULTIPAROUS (*n* = 165)		
Variable	Case Group	Control Group		Case Group	Control Group	
	*n* (%)	*n* (%)	*p*-Value	*n* (%)	*n* (%)	*p*-Value
Type of birth						
Vaginal	110 (82.1)	112 (70.9)		43 (97.7)	111 (91.7)	
Caesarean	24 (17.9)	46 (29.1)	0.027	1 (2.3)	10 (8.3)	0.291
Type of vaginal birth						
Normal	81 (73.6)	83 (74.1)		38 (88.4)	106 (95.5)	
Instrumented	29 (26.4)	29 (25.9)	0.936	5 (11.6)	5 (4.5)	0.143
3rd–4th-degree tears	4 (3)	4 (2.5)	0.990	0 (0)	5 (4.1)	0.346
Episiotomy	59 (44)	77 (48.7)	0.422	12 (27.3)	35 (28.9)	0.835
Apgar ≤7						
1 min ^a^	10 (8.1)	26 (20.6)	0.010	3 (7.3)	5 (5.3)	0.697
5 min ^b^	1 (0.8)	5 (3.2)	0.227	0 (0)	1 (0.8)	0.990
Umbilical cord pH						
pH < 7.20 ^c^	9 (8.7)	32 (21.2)	0.011	5 (14.7)	15 (13.4)	0.783
Mean pH ^d^	7.30 ± 0.1	7.25 ± 0.1	0.006	7.30 ± 0.1	7.28 ± 0.1	0.451
Neonatal resuscitation ^e^	5 (4)	22 (15.9)	0.008	2 (5.1)	2 (1.8)	0.283

^a^*n* = 386, ^b^ *n* = 453, ^c^ *n* = 401, ^d^ mean ± SD, ^e^ *n* = 412; Significance level obtained by chi-square test and Student’s *t*-test; Significance *p*-values adjusted by Fisher´s exact test.

## Data Availability

The data presented in this study are available on request from the corresponding author.
